# Supplementary material for: Synergy between Wsp1 and Dip1 may initiate assembly of endocytic actin networks
Source: eLife. 2020 Nov 12;9:e60419. doi: 10.7554/eLife.60419 (PMC7707826; doi:10.7554/eLife.60419)
Supplement: Supplementary file 1. — The best-fit values for the maximum polymerization rate at saturation (Max. Poly. Ratesat) and the concentration of Dip1 (µM) needed to reach half-saturated max polymerization rate for Dip1 alone or in the presence of Wsp1-VCA, GST-Wsp1-VCA, or Wsp1-CA. Data were fit to the following equation: Max poly rate = (max poly ratemax× [Dip1])/(K1/2 + [Dip1]) + y-intercept. The y-intercept was set as the maximum polymerization rate in the absence of Dip1 for each condition. [file elife-60419-supp1.docx]

|  | **Dip1 Alone** | **Dip1 + Wsp1-VCA** | **Dip1 + GST-VCA** | **Dip1 + Wsp1-CA** |
| --- | --- | --- | --- | --- |
| **Best-fit values** |  |  |  |  |
| Max. Poly. Rate_sat_ | 19.8 | 28.6 | 11.8 | 17.1 |
| K_1/2_ | 4.2 | 0.48 | 0.57 | 2.9 |
| **Std. Error** |  |  |  |  |
| Max. Poly. Rate_sat_ | 0.42 | 0.89 | 0.33 | 0.69 |
| K_1/2_ | 0.38 | 0.08 | 0.08 | 0.54 |
| **95% Confidence Intervals** |  |  |  |  |
| Max. Poly. Rate_sat_ | 18.8 to 20.7 | 26.6 to 30.6 | 11.0 to 12.5 | 15.6 to 18.7 |
| K_1/2_ | 3.4 to 5.1 | 0.30 to 0.65 | 0.38 to 0.76 | 1.7 to 4.1 |
| **Goodness of Fit** |  |  |  |  |
| R² | 0.997 | 0.977 | 0.984 | 0.985 |

Supplementary Table 1
