# Supplementary material for: Synergy between Wsp1 and Dip1 may initiate assembly of endocytic actin networks
Source: eLife. 2020 Nov 12;9:e60419. doi: 10.7554/eLife.60419 (PMC7707826; doi:10.7554/eLife.60419)
Supplement: Supplementary file 2. — Rate constants were set based on previous published values or optimization of parameters using simple models generated in this study, as described in the main text and methods or indicated by the given reference. Units for kon are M−1s−1 except for reactions 4, 5, 6, and 11 where the units are s−1. * indicates that a range of values for these parameters can yield a good fit to the experimental data (see Figure 3—figure supplement 2). [file elife-60419-supp2.docx]

Supplementary Table 2

| **Reaction #** | **Description** | **k_on_** | **k_off_ (s^-1^)** | **K_D_ (µM)** | **Reference** |
| --- | --- | --- | --- | --- | --- |
| 1 | Actin dimerization | 1.16 x 10^7^ | 5.88 x 10^4^ | 5.07 x 10^3^ | This Study |
| 2 | Actin trimerization | 1.16 x 10^7^ | 9.81 x 10^3^ | 846 | This Study |
| 3 | Actin tetramerization | 1.16 x 10^7^ | 96.9 | 8.36 | This Study |
| 4 | Actin dimer nucleation | 1.13 x 10^-4^ |  |  | This Study |
| 5 | Actin trimer nucleation | 1.41 x 10^-3^ |  |  | This Study |
| 6 | Actin tetramer nucleation | 5.86 x 10^-2^ |  |  | This Study |
| 7 | Barbed end elongation | 1.16 x 10^7^ | 1.4 | 0.12 | Pollard 1986 |
| 8 | Arp2/3 binds actin filament | 150 | 0.001 | 6.67 | Beltzner 2007 |
| 9 | Dip1 binds Arp2/3 | 1 x 10^6^ | 9.9 | 9.9 | This Study |
| 10 | Actin monomer binds Dip1-Arp2/3 nucleus | 1.16 x 10^7^ | 95200* | 8.21 x 10^3^ | This Study |
| 11 | Dip-bound Arp2/3 nucleation | 1.8* |  |  | This Study |
