# Supplementary material for: Synergy between Wsp1 and Dip1 may initiate assembly of endocytic actin networks
Source: eLife. 2020 Nov 12;9:e60419. doi: 10.7554/eLife.60419 (PMC7707826; doi:10.7554/eLife.60419)
Supplement: Supplementary file 3. — The table depicts a subset of Supplementary file 2 reaction rate constants that were floated in the one monomer-binding model to determine the best fit parameter values for datasets with Arp2/3 complex and Dip1 with or without Wsp1. Units for kon are M−1s−1 except for reaction 11, where units are s−1. * indicates that a range of values can yield a good fit to the experimental data (see Figure 3—figure supplement 2). [file elife-60419-supp3.docx]

Supplementary Table 3

|  | | **Dip1 Alone Dataset** | | **Dip1 + Wsp1 Dataset** | |
| --- | --- | --- | --- | --- | --- |
| Reaction # | Description | k_on_ | k_off_ (s^-1^) | k_on_ | k_off_ (s^-1^) |
| 9 | Dip1 binds Arp2/3 | 1 x 10^6^ | 9.9 | 1 x 10^6^ | 2.8 |
| 10 | Actin monomer binds Dip1-Arp2/3 nucleus | 1.16 x 10^7^ | 95200* | 1.16 x 10^7^ | 19.6 |
| 11 | Dip-bound Arp2/3 nucleation | 1.8* |  | 0.0053 |  |
