# Supplementary material for: Synergy between Wsp1 and Dip1 may initiate assembly of endocytic actin networks
Source: eLife. 2020 Nov 12;9:e60419. doi: 10.7554/eLife.60419 (PMC7707826; doi:10.7554/eLife.60419)
Supplement: Supplementary file 4. [file elife-60419-supp4.docx]

| ***S. pombe*** | |
| --- | --- |
| **Strain** | **Genotype** |
| VS698-A1 | *h^+^ ade6-M210 leu1-32 ura4-D18 his3-D1 fim1-mEGFP-kanMX6* |
| VS875-1D | *h^+^ ade6-216 leu1-32 ura4-D18 his3-D1 fim1-mGFP-kanMX6 wsp1∆CA-Tadh1-natMX6* |
| VS1981-2D | *h^-^ ade6-M210 leu1-32 ura4-D18 his3-D1 dip1∆::ura4+ fim1-mEGFP-kanMX6* |
| VS2053-2A | *h (n.d.) ade6-M210 leu1-32 ura4-D18 his3-D1 wsp1∆CA-Tadh1-natMX6 dip1∆::ura4+*  *fim1-mEGFP-kanMX6* |
| ***S. cerevisiae*** | |
| ScBN021 | *MATα, ura3-52, his3-∆200, leu2-3, lys2-801, trp1-901, ∆arp2::TRP1 ∆arp3::HIS3 pBN09::LEU2 pBN03::LYS2* |
